# Validation of the Idylla GeneFusion assay to detect fusions and *MET* exon-skipping in non-small cell lung cancers

**DOI:** 10.1038/s41598-023-39749-4

**Published:** 2023-08-09

**Authors:** Pauline Gilson, Celso Pouget, Richard Belmonte, Smahane Fadil, Jessica Demange, Marie Rouyer, Julien Lacour, Margaux Betz, Julie Dardare, Andréa Witz, Jean-Louis Merlin, Alexandre Harlé

**Affiliations:** 1 Institut de Cancérologie de Lorraine, CNRS UMR 7039 CRAN, Université de Lorraine, 6 Avenue de Bourgogne, CS 30519, 54519 Vandœuvre-lès-Nancy Cedex, France; 2 Service de Biologie Moléculaire des Tumeurs, Département de Biopathologie, Institut de Cancérologie de Lorraine/CHRU Nancy, Rue du Morvan, 54511 Vandœuvre-lès-Nancy Cedex, France; 3 Service d’Anatomocytopathologie, Département de Biopathologie, Institut de Cancérologie de Lorraine/CHRU Nancy, Rue du Morvan, 54511 Vandœuvre-lès-Nancy Cedex, France

**Keywords:** Non-small-cell lung cancer, Cancer genomics

## Abstract

Gene fusions and *MET* exon skipping drive oncogenesis in 8–9% and 3% of non-small cell lung cancers (NSCLC) respectively. Their detection are essential for the management of patients since they confer sensitivity to specific targeted therapies with significant clinical benefit over conventional chemotherapy. Immunohistochemistry (IHC) and fluorescent in situ hybridization (FISH) account for historical reference techniques however molecular-based technologies (RNA-based sequencing and RT-PCR) are emerging as alternative or complementary methods. Here, we evaluated the analytical performance of the fully-automated RT-PCR Idylla GeneFusion assay compared to reference methods using 35 fixed NSCLC samples. Idylla demonstrated overall agreement, sensitivity and specificity of 100% compared to RNASeq. Interestingly, it succeeded in retrieving 10 out of 11 samples with inconclusive results due to insufficient RNA quality for sequencing. Idylla showed an overall agreement, sensitivity and specificity of 90.32%, 91.67% and 89.47% compared to IHC/FISH respectively. Using commercial standards, the limit of detection of the Idylla system for the most frequent fusions and exon skipping ranges between 5 and 10 ng RNA input. These results support that the Idylla assay is a reliable and rapid option for the detection of these alterations, however a particular attention is needed for the interpretation of the expression imbalance.

## Introduction

Lung cancer represents the second most diagnosed cancer and the first cause of death worldwide, contributing for 11.4% of all new diagnosed cancers and 18% of all cancer-related deaths^1^. Despite improvements in diagnostic procedures, about 49% of patients are diagnosed with distant stage lung malignancies, associated with a limited number of therapeutic strategies and a poor prognosis [with a 5-year survival rate of 8.2% according to Surveillance, Epidemiology, and End Results (SEER) cancer data]. Non-small cell lung cancers (NSCLC) represent almost 85% of all lung cancer cases and comprise different histological subtypes, including adenocarcinomas, squamous cell lung carcinomas and large cell carcinomas^2–4^. Major efforts have been made in the last decades to better decipher NSCLC carcinogenesis and develop molecular-specific therapeutic options. The identification of gene amplifications, mutations and gene fusions as major driver alterations in NSCLC has led to the development of specific inhibitors that have revolutionized the clinical outcomes of patients. In 2019, more than 30% of NSCLC displayed oncogenic alterations that make patients eligible to targeted therapies^5^.

Besides the determination of the PDL1 status, experts from the European Society for Medical Oncology (ESMO) currently recommend the molecular genotyping of *EGFR, KRAS* and *BRAF* genes for all patients with advanced non-squamous cell carcinoma considering their major implications to identify patients eligible to targeted treatment (ESCAT scores IA-IB)^6^. Gene fusion assessment of *ALK, ROS1, RET* and *NTRK* gene fusions (ESCAT scores IA-IB-IC) and *MET* exon 14 skipping (ESCAT score IB) is also recommended^6^.

Gene fusions result from chromosomal rearrangements (deletions, inversions, translocations or duplications within the same chromosome or between 2 different chromosomes) that lead to the fusion of 2 normally independent genes. Gene fusions can be found in 8–9% of NSCLC, including 3–7% of cases with ALK fusions, 0.9–2.6% with ROS1 fusions, 1–2% with RET fusions and 0.1–0.17% with NTRK fusions^7–11^. Gene fusions predominantly occur in adenocarcinoma subtype (12% of cases) and in metastatic stage disease^9^. The number of gene partners implied in fusions are highly variable with up to 24 fusion partners identified in ROS-rearranged NSCLC, 48 in RET-rearranged NSCLC and 92 in ALK-rearranged NSCLC^12–14^. All these gene fusions give rise to cancer susceptibility to different specific targeted therapies^6^.

*MET* exon 14 skipping occurs in almost 3% of NSCLC^15^, particularly in patients over 70 years with a smoking history and/or pleomorphic carcinoma or adenosquamous cell carcinoma subtypes^15^. These events can originate from more than 500 different molecular alterations located at branch sites, poly-pyrimidine tract, splice acceptor or donor sites of the *MET* gene that impacts RNA splicing, induces loss of the MET protein juxtamembrane domain and enhances MET oncogenic signalling pathways^15^. Clinical data demonstrated substantial benefit to use crizotinib multi-kinase inhibitor in advanced NSCLC patients with MET exon 14 skipping^16^. More recently, two MET kinase-specific inhibitors (capmatinib and tepotinib) have received US Food and Drug Administration (FDA) approval based on overall response rate and duration of response in patients with metastatic NSCLC harbouring *MET* exon 14 skipping in GEOMETRY mono-1 and VISION trials respectively^17, 18^.

Standard reference methods for molecular status determination in NSCLC include immunohistochemistry (IHC), Fluorescent In-Situ Hybridization (FISH) and molecular-based approaches such as RT-PCR or RNA sequencing (RNASeq). These methods are complementary options with distinct benefits and limitations. IHC can reveal aberrant protein expression caused by rearrangements. IHC has the advantage to be implemented and automated in most clinical laboratories and give rapid results with minimal costs that makes it the screening method of choice for the detection of major gene fusions. Moreover, a wide panel of antibodies covering molecular targets are now available for IHC testing. However, IHC is submitted to subjective interpretations, it can lead to inconclusive results due to preanalytical factors and its limit of sensitivity is quite high (15%). Moreover, fusion partners and precise breakpoints cannot be determined and several studies reported a lack of reliability to identify *MET* exon skipping or *RET* fusions by IHC^19–22^. FISH commonly accounts for a complementary approach to confirm doubtful IHC results or a screening tool when no specific and reliable IHC assay is available. FISH is commonly performed using break-apart probes that allow the detection of rearrangements whatever the fusion partner. Nevertheless, FISH suffers from prolonged technical and reading times and requires specific infrastructure and expertise. FISH can also miss rare cases of cryptic or complex chromosomal rearrangements and the quality of the slides strongly depends on the technical procedures used. Conventional RT-PCR assays look for fusion transcript variants at the RNA level with high sensitivity but these targeted approaches require previous diagnosis hypotheses about the 2 fusion partners and the exact breakpoints to select the appropriate probes and primers to use and offer only limited multiplexing possibilities. Sequencing methods represents broader approaches that cover a large number of gene fusions in one assay or can even detect all fusions without previous knowledge of the fusion partners depending on the panel design. RNA-based sequencing methods notably display better sensitivities and specificities than IHC and FISH, however they are time-consuming and cost-consuming.

The Idylla system (Biocartis, Mechelen, Belgium) is a novel fully automated RT-qPCR-based molecular diagnostics system that can identify a panel of specific gene fusions (including 16 specific *ALK* fusions, 13 *ROS1* fusions, 7 *RET* fusions and *MET* exon 14 skipping) as well as expression imbalance (in *ALK, ROS1, RET, NTRK1, NTRK2* and *NTRK3* genes) without pre-analytical RNA extraction^23^. In this retrospective study, we performed the evaluation of the Idylla GeneFusion system for the multiplex detection of *ALK, ROS1, RET, NTRK1, NTRK2, NTRK3* fusions and *MET* exon 14 skipping in 35 clinical formalin-fixed paraffin-embedded (FFPE) specimens from NSCLC patients using IHC/FISH and RNASeq as the gold standards. This included the analysis of 11 clinical samples that did not reach RNA quality requirements for RNASeq. We also determined the limit of detection of the cartridges for the detection of the most frequent gene fusions and exon skipping events using two different commercial controls.

## Results

### Evaluation of the Idylla GeneFusion assays to detect gene fusions and *MET* exon 14 skipping compared to standard reference techniques

We evaluated the analytical performance of the Idylla GeneFusion assay by considering their ability to detect either specific alterations in *ALK, ROS1, RET* or *MET* genes or expression imbalance in *ALK, ROS1, RET, NTRK1, NTRK2* or *NTRK3* genes (that could testify the presence of unknown fusions). The specific alterations covered by the Idylla GeneFusion assay are detailed in Supplementary Table S1.

The Idylla GeneFusion assay gave 94.29% of valid results for the detection of specific alterations. Only 2 samples (#5 and #34) out of the 35 analysed were found inconclusive by Idylla while results for 2 and 11 samples were previously found inconclusive by IHC/FISH and RNASeq respectively (Table 1). A total of 23 samples gave valid results by both RNASeq and Idylla techniques. All of the 23 cases were found concordant between RNASeq and Idylla GeneFusion assay for the detection of specific alterations, making an overall agreement of 100.00% for the Idylla GeneFusion assay using RNASeq as the gold standard. The sensitivity and specificity of the Idylla GeneFusion assay for the detection of specific alterations were 100.00% (18/18) and 100.00% (5/5) respectively using RNASeq as the reference method (Table 2). Positive and negative predictive values of the Idylla assay were 100.00% (18/18) and 100.00% (5/5) respectively. Interestingly, among the 11 FFPE samples (#25-#35) that were found inconclusive by RNASeq due to insufficient RNA quality, the Idylla GeneFusion assay was able to retrieve ten samples (#25-#33 and #35, 90.91% of cases). Those notably included two samples (#30 and #31) with actionable alterations detected by Idylla that could benefit from targeted therapies. Considering IHC and FISH as the gold standards, the overall agreement, sensitivity and specificity of the Idylla GeneFusion assay for the detection of specific alterations were 90.32% (28/31 samples with valid results), 91.67% (11/12) and 89.47% (17/19) respectively (Table 2). Positive and negative predictive values of the Idylla GeneFusion assay were 84.62% (11/13) and 94.44% (17/18) respectively.

**Table 1:** *ALK, ROS1, RET* and *MET* status obtained by IHC, FISH, RNASeq and Idylla GeneFusion assays.

ID sample	Reference methods		Idylla				
	RNASeq	IHC and FISH	Results for specific alterations	Results for expression imbalance	Delay between Idylla assay and sampling (months)	Cq of RNA controls	Cq of DNA controls
1	–	–	–	–	0	25.1	24.7
2	–	–	–	ALK+	0	25.7	26.5
3	–	–	–	–	0	26.5	29.4
4	–	–	–	–	0	24.7	26.8
5	–	–	NC	NC	0	N/A	26.2
6	–	ROS1 +	–	–	19	25.2	25.8
7	ALK+	ALK+	ALK+	ALK+	13	27.2	28
8	ALK+	ALK+	ALK+	ALK+	1	25.1	26
9	ALK+	– (borderline ALK FISH results)	ALK+	ALK+	3	27.6	28.1
10	ALK+	ALK+	ALK+	ALK+ NTRK2 NC	0	29.6	31.6
11	ALK+	ALK+	ALK+	ALK+	3	27.9	31.3
12	ALK+	ALK+	ALK+	ALK+	0	27.8	31.4
13	ALK+	– (discordant results between ALK IHC and FISH)	ALK+	ALK+	1	24.3	26.2
14	ALK+	ALK+	ALK+	ALK+ NTRK2 I NTRK3 I	2	28.2	30.3
15	ALK+	ALK+	ALK+	ALK+	9	28.4	30.8
16	ALK+	ALK+	ALK+	ALK+ NTRK1+	1	26.5	29.4
17	ROS1+	ROS1+	ROS1+	ROS1+ NTRK2 NC NTRK3 NC	1	28.1	29
18	ROS1+	ROS1+	ROS1+	ROS1+ ALK I NTRK2 I	7	30.6	32.5
19	RET+	–	RET+	RET+ ALK+	3	26.4	29.5
20	MET+	–	MET+	–	4	25.6	25
21	MET+	–	MET+	–	5	25.9	27.2
22	MET+	ALK− and ROS1 NC	MET+	–	0	27.6	31.5
23	MET+	–	MET+	–	2	25.8	26.7
24	Low sequencing quality but reasonable suspicion of MET+	–	MET +	–	1	29.5	29.4
25	NC	–	–	ALK NC	1	28.5	23.8
26	NC	–	–	–	6	27.4	29.6
27	NC	–	–	–	1	25.7	27.9
28	NC	–	–	–	2	25.3	27.3
29	NC	–	–	ALK+	1	27.5	29.3
30	NC	ALK− and ROS1 NC	ROS1+	ROS1+	48	28.5	29.1
31	NC	ALK+	ALK+	ALK+	19	28.1	29.4
32	NC	–	–	–	3	28.3	32
33	NC	–	–	ALK NC ROS1 NC RET NC NTRK1 NC NTRK3 NC	7	35.7	33.3
34	NC	–	NC	NC	0	N/A	N/A
35	NC	–	–	ALK I RET I NTRK1 I NTRK2 I NTRK3 I	12	35.9	34.4

+: presence of an alteration; −: absence of alteration, *FISH* fluorescent in-situ hybridization; *I* indeterminate, *IHC* immunohistochemistry, *N/A* not available, *NC* inconclusive, *RNASeq* RNA-based sequencing.

**Table 2:** Concordance between Idylla, RNASeq and IHC/FISH results.

	No. of cases with valid results for both techniques	No. of cases with alteration detected by both methods (+ / +)	No. of cases with no alteration detected by both methods (−/−)	No. of cases with alteration only detected by the tested method (+ /−)	No. of cases with alteration only detected by the reference method (−/ +)	OA (%)	Se (%) [95% CI]	Sp (%) [95% CI]	PPV (%)	NPV (%)
Idylla specific fusions module vs RNASeq*	23	18	5	0	0	100.00%	100.00% [82.41; 100.00]	100.00% [56.55; 100.00]	100.00%	100.00%
Idylla specific fusions module vs IHC/FISH*	31	11	17	2	1	90.32%	91.67% [64.61; 98.51]	89.47% [67.20; 96.90]	84.62%	94.44%
IHC/FISH vs RNASeq*	23	10	10	1	2	86.95%	83.33% [55.20; 95.30]	90.91% [62.26; 98.38]	90.91%	83.33%
Idylla expression imbalance module vs RNASeq*	23	13	9	1	0	95.65%	100.00% [77.19; 100.00]	90.00% [59.58; 98.21]	92.86%	100.00%

The asterisk indicate the technique set as reference for the determination of the overall agreement (OA), sensitivity (Se), specificity (Sp), positive predictive value (PPV) and negative predictive value (NPV) of the tested method. The 95% confidence intervals for sensitivity and specificity are indicated within square brackets.

+: presence of an alteration; −: absence of alteration, *FISH* fluorescent in-situ hybridization, *IHC* immunohistochemistry, *No.* number, *NPV* negative predicate value, *OA* overall agreement, *PPV* positive predicate value, *RNASeq* RNA-based sequencing, *Se* sensitivity, *Sp* specificity, *vs* versus.

Concerning the detection of unknown fusions using the expression imbalance module, the Idylla GeneFusion assay yielded invalid results for all genes in 2 out of 35 samples (#5 and #34) and gave only partial results in 7 out of the 35 samples analysed (#10, #14, #17, #18, #25, #33 and #35). In the samples analysed, all specific *ALK, ROS* and *RET* fusions detected by Idylla were associated with corresponding 5′–3′ gene expression imbalance (Table 1). Gene expression imbalance was also reported in two samples (#2 and #29) with no specific fusions detected by Idylla. Two samples harboured a specific gene fusion associated with expression imbalance of two different genes (#16 and #19). For these two cases, RNASeq only retrieve the specific gene fusion identified by Idylla. Considering RNASeq as the reference, the overall agreement, sensitivity and specificity of the Idylla GeneFusion assay for the detection of expression imbalance were 95.65% (22/23 samples with valid results), 100.00% (13/13) and 90.00% (9/10) respectively (Table 2). Positive and negative predictive values of the Idylla GeneFusion assay were 92.86% (13/14) and 100.00% (9/9) respectively. The expression imbalance module of the Idylla GeneFusion assay gave valid results for 7 out of the 11 clinical samples (#26 to #32) that were found uninterpretable by RNASeq.

### Determination of the limit of detection (LOD) of the Idylla GeneFusion assay

We determined the limit of detection (LOD) of the Idylla GeneFusion assay for the detection of *ALK, ROS1, RET, NTRK1, NTRK2, NTRK3* fusions and *MET* exon 14 skipping using different inputs of two commercial standards directly loaded into the Idylla GeneFusion cartridges. Using the SeraSeq commercial standard, the LOD was 5 ng for the detection *ALK* and *ROS1* specific gene fusions and *MET* exon 14 skipping and 7.5 ng for the detection of *RET* specific gene fusion (Table 3). An expression imbalance was observed in *ALK* and *NTRK3* genes until 5 ng of RNA as starting material while the analysis of expression imbalance was difficult to interpret for other genes mainly due to invalid PCR curves. Using the Horizon commercial standard, the LOD was 10 ng for the detection *ALK, ROS1* and *RET* specific gene fusions. An expression imbalance was reported until 10 ng input for *ALK* and *ROS1* genes and 15 ng for *RET* gene. The expression imbalance module indicated invalid PCR curves for *NTRK* genes resulting in indeterminate results.

**Table 3:** Limits of detection (LOD) of the Idylla GeneFusion assay for the detection of *ALK, ROS1* and *RET* specific gene fusions, *MET* exon 14 skipping as well as unknown gene fusions.

Commercial standard	Input (ng)	Cq of the RNA controls	Cq of the DNA control	Genomic alterations		Biological interpretation	Cq	∆Cq
SeraSeq	50	32	31.8	Specific alterations	ALK	Detected	31	–
					*ROS1*	Detected	30.9	–
					*RET*	Detected	31.6	–
					*MET* exon 14 skipping	Detected	30.8	–
				Expression imbalance	*ALK*	Detected	NA	0.2
					*ROS1*	Indeterminate	–	–
					*RET*	Indeterminate	–	–
					*NTRK1*	Indeterminate	–	–
					*NTRK2*	Not detected	31.6	5.8
					*NTRK3*	Detected	NA	–0.3
	30	32.3	32.4	Specific alterations	*ALK*	Detected	31.4	–
					*ROS1*	Detected	31.2	–
					*RET*	Detected	31.9	–
					*MET* exon 14 skipping	Detected	31.1	–
				Expression imbalance	*ALK*	Detected	NA	0.7
					*ROS1*	Indeterminate	–	–
					*RET*	Not detected	NA	5.8
					*NTRK1*	Indeterminate	–	–
					*NTRK2*	Not detected	32.3	5.8
					*NTRK3*	Detected	NA	−0.8
	15	35.2	34.2	Specific alterations	*ALK*	Detected	33.1	–
					*ROS1*	Detected	34.2	–
					*RET*	Detected	34.9	–
					*MET* exon 14 skipping	Detected	34.1	–
				Expression imbalance	*ALK*	Detected	NA	−0.3
					*ROS1*	Indeterminate	–	–
					*RET*	Indeterminate	–	–
					*NTRK1*	Indeterminate	–	–
					*NTRK2*	Indeterminate	–	–
					*NTRK3*	Detected	NA	−0.8
	7.5	33.2	32	Specific alterations	*ALK*	Detected	31.9	–
					*ROS1*	Detected	30.6	–
					*RET*	Detected	32.7	–
					*MET* exon 14 skipping	Detected	31.8	–
				Expression imbalance	*ALK*	Detected	NA	0.3
					*ROS1*	Indeterminate	–	–
					*RET*	Indeterminate	–	–
					*NTRK1*	Indeterminate	–	–
					*NTRK2*	Not detected	32.8	4.3
					*NTRK3*	Detected	NA	−0.6
	5	35.6	34.8	Specific alterations	*ALK*	Detected	34.6	–
					*ROS1*	Detected	34.8	–
					*RET*	Not detected	–	–
					*MET* exon 14 skipping	Detected	35.1	–
				Expression imbalance	*ALK*	Detected	NA	0.5
					*ROS1*	Indeterminate	–	–
					*RET*	Indeterminate	–	–
					*NTRK1*	Indeterminate	–	–
					*NTRK2*	Indeterminate	–	–
					*NTRK3*	Detected	NA	−0.2
	3	not amplified	34.6	Invalid				
Horizon	15	34.6	35	Specific alterations	*ALK*	Detected	34.5	–
					*ROS1*	Detected	34.6	–
					*RET*	Detected	32.2	–
					*MET* exon 14 skipping	Not detected	–	–
				Expression imbalance	*ALK*	Detected	NA	0.7
					*ROS1*	Detected	38.5	−5
					*RET*	Detected	36.3	−1.9
					*NTRK1*	Not detected	37.8	−1
					*NTRK2*	Indeterminate	–	–
					*NTRK3*	Indeterminate	–	–
	10	34.9	34.9	Specific alterations	*ALK*	Detected	35.8	–
					*ROS1*	Detected	35.3	–
					*RET*	Detected	32.3	–
					*MET* exon 14 skipping	Not detected	–	–
				Expression imbalance	*ALK*	Detected	NA	1.1
					*ROS1*	Detected	37.5	−3.2
					*RET*	Not detected	36	−1.2
					*NTRK1*	Indeterminate	–	–
					*NTRK2*	Indeterminate	–	–
					*NTRK3*	indeterminate	–	–
	7.5	Not amplified	35.7	Invalid				

Interpretation of the Idylla results: the Idylla analysis is considered inconclusive if RNA control curves are invalid, and the DNa control Cq is determined to assess RNA degradation; specific fusion is found “detected” if the PCR curve is valid and the Cq appears within a predefined range; *MET* exon 14 skipping is found “detected” if the corresponding PCR curve is valid, the Cq is within a predefined range and the difference between METex14 and MET wild-type Cq (ΔCq) is below a predefined threshold; the detection of an “expression imbalance” indicates a difference between 3' end and 5' end expression levels of the gene and should reflect the presence of an unknown gene fusion that need to be confirmed by complementary techniques (IHC, FISH or NGS); the analysis of expression imbalance requires the validity of the 3' curve, otherwise the result is qualified as “indetermined”. If the 3′ and 5′ curves are valid, the expression imbalance is considered “detected” if the ΔCq value (i.e. the difference between 3′ and 5′ Cq) is below a predefined (5′ Cq value-dependent) threshold. If the 3′ curve is valid while the 5′ curve is invalid, the ΔCq value is calculated between 3′ and RNA control curves and the expression imbalance is found “detected” if the ΔCq value is below a predefined threshold

*Cq* cycle quantification, *NA* not applicable.

## Discussion

**Figure 1:**
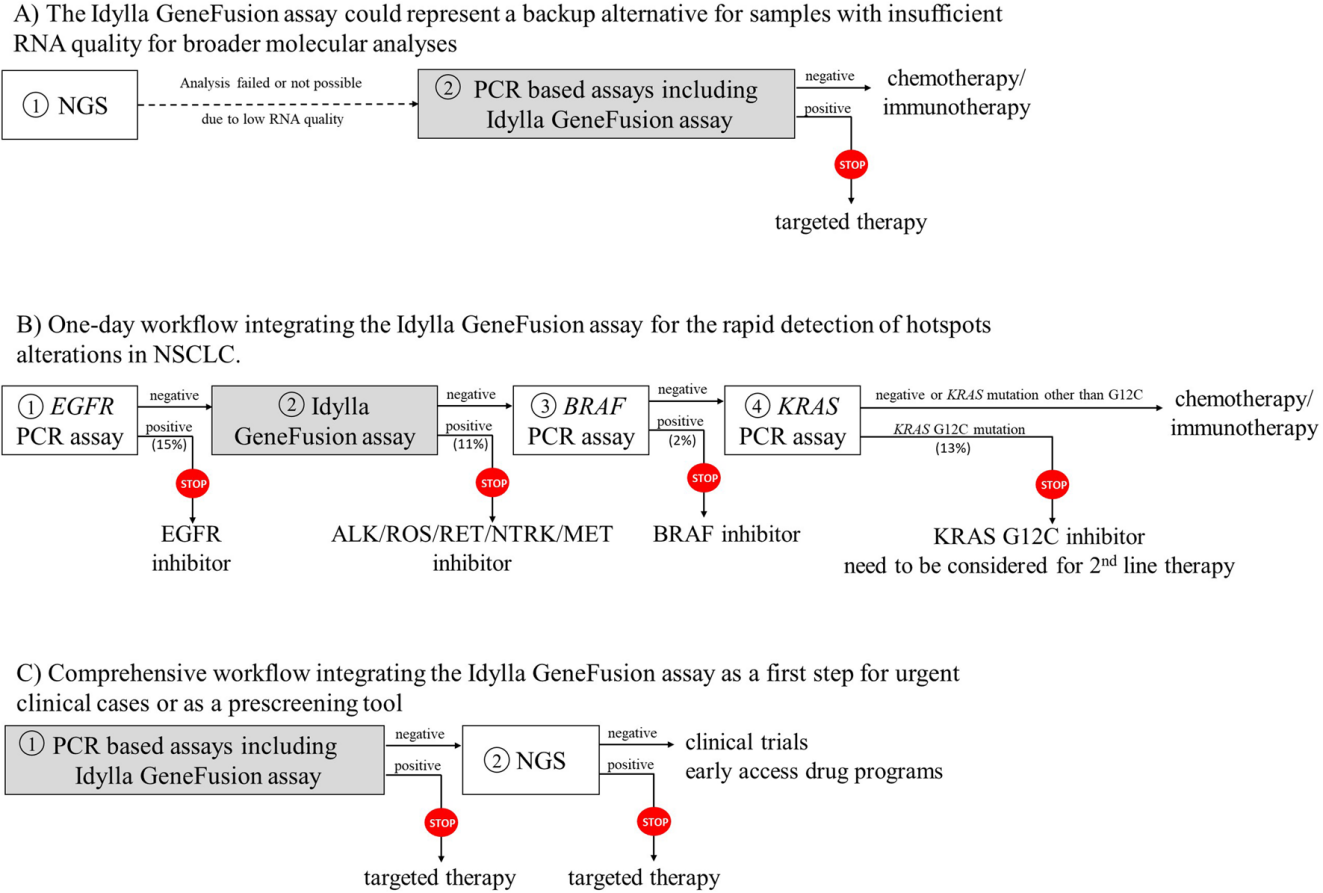
Proposition of laboratory workflows integrating the Idylla GeneFusion assay.

Here, we performed the validation of the Idylla GeneFusion assay for the detection of the most frequent gene fusions and exon skipping observed in NSCLC. Using 35 fixed samples previously characterized by IHC/FISH and RNASeq, we showed that the Idylla results were comparable with those obtained from reference testing methods. Notably, a 100% overall agreement was observed between RNASeq and Idylla for the detection of specific gene fusions. A lower overall agreement was observed between Idylla and IHC/FISH methods, albeit comparable to that reported between RNASeq and IHC/FISH. Interestingly, Idylla showed ability to analyze problematic samples that were previously rejected for RNASeq due to low RNA quality. Thus, the Idylla system meets the clinical need to rescue samples that appeared suboptimal for other molecular analyses and avoid rebiopsy (Fig. 1A). Our evaluation of the Idylla cartridges using commercial RNA standards highlighted the fact that extracted RNA can be directly pipetted into the Idylla GeneFusion cartridges for analysis, as previously observed for other Idylla PCR-based assays^24–29^. This could be of particular interest for samples rejected for NGS analysis due to low RNA quality, as archival extracted RNA could be reused for Idylla assay instead of collecting supplementary FFPE sections.

Moreover, Idylla has the advantages of being easily implemented into clinical laboratories compared to RNASeq and FISH. It can be performed on-demand as minimal hands-on-time is required and batching samples is not necessary to launch the analysis. It offers a reduced turnaround time (180 min), thus appearing as a suitable molecular-based option for clinical cases with urgent treatment decision making.

In return, the Idylla GeneFusion assay has some limitations that need to be taken into account. Considering its limited scope, the Idylla GeneFusion assay should be combined with other approaches in order to fulfill all molecular testing guidelines in newly diagnosed advanced NSCLC^6^. As proposed by other research groups, the Idylla GeneFusion assay and other PCR-based assays could be integrated into a 1-day laboratory workflow for the detection of hotspot *KRAS, EGFR* and *BRAF* mutations as well as major *ALK, ROS1, RET* and *NTRK* gene fusions and *MET* exon 14 skipping events^30^. Given that these alterations of high clinical impact are theoretically mutually exclusive, sequential analyses could be proposed based on the indications of the different targeted therapies and the frequencies of the alterations observed in NSCLC (Fig. 1B). In this workflow, treatment decision can be made in a minimum delay time and molecular analyses can guide the prescription of currently approved targeted therapies. A second and more comprehensive laboratory workflow could involve PCR and/or Idylla GeneFusion assay to rapidly interrogate hotspots alterations followed by a broader NGS-based analysis (Fig. 1C). Such workflow could help for treatment decisions in urgent clinical cases without affecting patients’ access to early drug access programs or clinical trials. In this workflow, the Idylla GeneFusion assay could also represent a prescreening tool prior to more expensive NGS analyses. Nevertheless, both proposed workflows are only acceptable for clinical specimens with sufficient tumour material to perform multiple analyses.

It should be noted that the Idylla system cannot specify the molecular partner involved in gene fusions. This information could have clinical stakes given the fact that several studies recently reported differential responses to targeted therapies depending on the partner type^31–33^.

Moreover, the Idylla assay was not designed to identify resistance mechanisms that can emerge upon exposure ALK, RET, ROS1 or NTRK inhibitors making such analysis inadequate to evaluate disease progression and guide treatment modification.

Besides the detection of specific gene fusions, the Idylla GeneFusion cartridges were designed to detect unknown fusions with atypical breakpoints or novel fusion partners by means of 5′–3′ imbalance analysis. However, our study on clinical samples highlighted four false positive results with the expression imbalance module compared to gold standards. Moreover, inconclusive results were obtained for all genes studied or a small subset of them in almost 26% of samples, thus requiring other analyses to conclude. The expression imbalance module also appeared not adapted for the analysis of the two commercial RNA standards as indeterminate results frequently occurred whatever the RNA input tested. A particular attention is then needed for the interpretation of 5′–3′ expression imbalance results.

Due to the very low frequency of *NTRK* gene fusions in NSCLC (around 0.1% of all cases) ^7^, our study lacks from the analysis of clinical NSCLC samples with characterized *NTRK* fusions. Despite the use of commercial RNA reference standards with known *NTRK* fusions, we were not able to evaluate the Idylla cartridges for the detection of *NTRK* gene fusions due to partial results obtained. Other research groups recently succeeded in estimating the performance of the Idylla GeneFusion assay for the detection of *NTRK* fusions using a limited number of extrapulmonary tumour samples or through multicenter studies^30, 34–36^. They notably reported a limited sensitivity of the cartridges for *NTRK* fusions compared to other gene fusions, probably due to the analysis by expression imbalance only without fusion specific detection method. Another study evaluating the interest of the Idylla GeneFusion cartridges to detect NTRK fusions in pan-cancer samples highlighted some false positive and false negative results^37^. However, these incorrect results were more frequently observed in extrathoracic tumours, for which the cartridges were not designed for. Indeed, NTRK higher expression levels were reported in some cancer types (particularly in central nervous system tumours) compared to pulmonary tumours that can affect the analysis by expression imbalance.

To conclude, the Idylla GeneFusion assay appears as a fast and reliable alternative to reference methods to detect gene fusions. Due to its minimal hands-on-time and low turnaround time, it could be easily integrated into laboratory workflows in order to give more rapid tumour molecular profiles or retrieve samples rejected by other molecular techniques. A careful interpretation is however needed for the determination of gene fusions using the expression imbalance module.

## Material and methods

### Sample selection

The research was conducted on 35 formalin-fixed paraffin-embedded (FFPE) tumour samples from NSCLC patients that were retrospectively selected among the biological collection of Lorraine Cancer Institute (ICL, Vandœuvre-lès-Nancy, France) and Nancy University Hospital Center (CHRUN, Vandœuvre-lès-Nancy, France). All samples were obtained during standard clinical practice based on the research of clinically actionable mutations and fusions in NSCLC to determine appropriate treatment. Notably, samples were analysed using immunohistochemistry (IHC), fluorescent *in-situ* hybridization (FISH) and RNA-based sequencing (RNASeq) according to current expert recommendations. A written informed consent was obtained from all patients enrolled in this study for the use of their biological specimens for research purposes. Data collected were anonymised at the time of inclusion. The clinical characteristics of the patients are detailed in Table 4. This study was approved by the ethical and scientific committee of Lorraine Cancer Institute and all patients gave their consent for the use of their clinical samples. All experiments were performed according to the relevant guidelines and regulations. All results were interpreted by senior biologists and pathologists who were blinded to the previous results.

**Table 4:** Clinical characteristics of the 35 NSCLC patients enrolled in this study.

Sex	
Female	18 (51.4%)
Male	17 (48.6%)
Age at diagnosis (years)	
Median [interquartile range]	67 [60; 71]
Tumour histological subtype (according to the 2015 World Health Organization Classification of Lung Tumors) ^4^	
Pleomorphic carcinoma	2 (5.71%)
Adenocarcinoma	33 (94.29%)
Lepidic	1 (2.86%)
Solid	10 (28.57%)
Enteric	2 (5.71%)
Acinar	13 (37.14%)
Invasive mucinous	2 (5.71%)
Invasive nonmucinous	5 (14.29%)
Smoking habit	
Non-smoker	8 (22.9%)
Former smoker	15 (42.9%)
Current smoker	4 (11.4%)
Data not provided	8 (22.9%)
Tumour stage	
Non-metastatic	24 (68.6%)
Metastatic (stage IV)	11 (31.4%)

### Immunohistochemistry (IHC)

IHC assays were performed according to the routine practices of the ICL-CHRUN pathology lab. Briefly, immunohistochemistry staining of ALK and ROS1 proteins was performed on 5 µm-thick unstained FFPE sections using the automated Ventana BenchMark ULTRA IHC/ISH system (Roche Diagnostics, Tucson, AZ, USA). Anti-ALK antibody (5A4 clone, dilution 1:100, Abcam, Cambridge, UK) and anti-ROS1 antibody (SP384 clone, Roche Diagnostics) were used. Detection was performed using the OptiView DAB IHC Detection Kit (Roche Diagnostics). IHC staining patterns were checked for each antibody using positive and negative internal or external controls and slides were examined by a senior pathologist. A score was established (score 0, 1+ , 2+ or 3+) depending on the percentage of stained cells and the staining intensity. Regarding ALK IHC assays, cases scored as 0 were considered negative, those with score 3+ were designed as ALK-positive and those with score 1+ or 2+ were defined equivocal and required a complementary FISH technique^38^. Regarding ROS1 IHC assays, cases scored as 0 were considered ROS1-negative while cases with positive scores should to be confirmed by FISH or molecular-based techniques according to expert consensus opinion^39^. Given the poor value of MET and RET IHC to screen *MET* exon 14 skipping and *RET* fusions, RNASeq was used as the standard reference method to detect these alterations^19–22^.

### Fluorescence in situ hybridization (FISH)

*ALK* and *ROS1* rearrangements were investigated by FISH in case of equivocal IHC results. Unstained FFPE tumour tissue sections were incubated with dual-colour ALK and ROS1 IQFISH Break-Apart Probes (Dako Omnis, Agilent, Santa Clara, CA, USA) using Dako Omnis instrument (Dako Omnis) following the manufacturer’s instructions. The slides were screened using Olympus BX51 epifluorescence microscope (Olympus Corporation, Tokyo, Japan). For interpretation of FISH status, at least 100 intact tumour nuclei cells were needed. Tumour samples were considered ALK- or ROS1-rearranged if more than 15% of tumour cells harboured split red and green signals and/or single fluorescent red signals^40, 41^. Otherwise, the samples were interpreted as FISH negative.

### RNA-based sequencing (RNASeq)

Targeted RNA-based sequencing was performed using the commercial FusionPlex Lung Panel (Integrated DNA technologies, Coralville, Iowa, USA). Besides the research of hotspot single nucleotide variations (SNVs) and small insertions-deletions (indels) in *ALK, BRAF, KRAS, EGFR, RET* and *ROS1* genes, the panel is designed to detect fusions in 13 genes including *ALK*, *BRAF*, *EGFR, FGFR1, FGFR2, FGFR3*, *MET*, *NRG1, NTRK1, NTRK2, NTRK3, RET* and *ROS1* (see details in Suppl. Table S2*)*. The enrichment method is based on anchored Multiplex PCR (AMP) technology that allows the detection of known and novel gene fusions without any prior knowledge of the fusion partners. Briefly, FFPE tumour tissue samples were macrodissected to obtain 5-µm thick sections with at least 10% tumour content. Total RNA was extracted using the RNeasy FFPE kit (Qiagen, Les Ulis, France) without adding DNAse during the process (DNA found in the sample serves as a measure of sequencing quality). Extracted RNA was quantified using the Qubit 3 fluorometer (Invitrogen, Thermo Fisher Scientific, Waltham, MA, USA) and the Qubit RNA HS Assay kit (Thermo Fisher Scientific). A total of 250 ng total RNA input was used for the preparation of the libraries. RNA was converted into cDNA by reverse transcription using random primers. After first strand cDNA synthesis, a real-time PCR-based assay was performed using the Archer PreSeq RNA quality control (QC) Assay (Pre-Seq Ct score) (Invitae Corporation) to assess RNA quality. Cases with Pre-Seq Ct score > 28 were qualified as having poor RNA quality. Double-stranded cDNA undergoes end repair, dA tailing and ligation using partially-functional molecular barcode (MBC) adapters that contain an universal primer binding site. The generated fragments were amplified by two-step PCR using gene-specific primers (GSPs) targeting the genes of interest and housekeeping genes, and universal primers complementary to MBC adapters. Libraries were quantified using the KAPA Universal Library Quantification kit (Kapa Biosystems, Potters Bar, UK), pooled at equimolar concentrations, and sequenced at 2× 150 base pairs using the MiSeq instrument (Illumina, San Diego, CA, USA). Raw data were processed using the Archer Analysis Software and version 6.2.7 pipeline (Integrated DNA technologies).

The Seraseq^®^ FFPE Tumor Fusion RNA v4 Reference Material (reference 0710-0496, LGC Seracare, Milford MA, USA) was used as a quality internal control for each run (see details on the specific alterations covered in Suppl. Table S3). Sequencing quality was assessed by the following metrics: a minimum of 500,000 total reads per sample, an on-target > 85%, a percentage of total RNA reads over the percentage of total DNA reads, a minimum of 20,000 reads and 30% reads for RNA unique fragments, an average unique RNA start sites per GSP2 control (Fusion QC score) > 10, a RNA median fragment length > 100 base pairs (Suppl. Table S4). The analysis of all these quality metrics is needed to determine the interpretability of the RNAseq data. Gene fusions were classified as “structural variations with strong evidence” if they have been already described as known fusions in the Archer database and/or if they meet the following criteria: > 3 unique start sites, > 5 unique reads and > 10% of reads supporting the event.

### Idylla GeneFusion assay

The fully-automated Idylla GeneFusion assay (reference A0121/6, Biocartis NV, Mechelen, Belgium) allows the detection of multiple gene fusions and exon-skipped transcripts in a single assay with less than 5 min hands-on-time. The single-use Idylla cartridges include all reagents on board to cover all steps from sample-to-result starting from FFPE tumour sections. The number of FFPE sections depends on the tissue area: one 5 µm-thick section is sufficient for tissue surface over 20 mm^2^, otherwise three sections are required. Macrodissected FFPE tumour sections with at least 10% tumour content were inserted into the cartridge and loaded into the Idylla instrument (Biocartis NV). The whole process lasts 180-min and comprises sample liquefaction, total nucleic acid extraction, reverse transcription of mRNA, real-time PCR amplification, fluorophore-based detection, automated data analysis and final report generation.

The Idylla GeneFusion assay is designed for the detection of specific *ALK, ROS1* and *RET* gene fusions and *MET* exon 14 skipping (Suppl. Table S1) as well as structural rearrangements without prior knowledge of the fusion partners by analysing the expression ratios between the 5′ and 3′ ends of *ALK, ROS1, RET, NTRK1, NTRK2* and *NTRK3* genes. The expression level of two RNA controls (ERCC3 and TMUB2) is evaluated in each sample to testify the correct execution of the whole process. The Cq of the RNA controls reflects the amount of amplifiable RNA in the sample and allows the analysis of expression imbalance when 5′ kinase expression is insufficient. An internal DNA control (KIF11) is also included in the cartridge and serves as an indicator of RNA degradation in cases of invalid results. The MET wild-type control helps to prevent incorrect calling due to MET overexpression.

### Molecular characteristics of the 35 tumour samples analysed

The 35 tumour specimens were collected from NSCLC patients between September 2017 (#30) and May 2022 (#16). The delay between Idylla analysis and tumour sampling ranged between 0 (#1 to #5, #9, #12, #22 and #34) and 48 months (#30) with a median of 2 months. The specimens consist in surgical resections (n = 21 samples, 60%), biopsies (n = 12 samples, 34.29%) or cytology samples (n = 2 samples, 5.71%) and tumour content varied from 10 to 85% with a median of 45% (Table 5). Out of the 35 samples analysed by RNASeq, 10 harboured an *ALK* gene fusion (#7 to #16), 2 had a *ROS1* gene fusion (#17 to #18), 1 had a *RET* fusion (#19), 5 had a *MET* exon 14 skipping (#20 to #24) and 6 had a negative status for *ALK, ROS1, RET, NTRK* and *MET* genes (#1 to #6). Eleven out of the 35 samples (#25 to #35) were found inconclusive by RNASeq due to the absence of alterations detected and the presence of non-satisfied quality criteria: Pre-Seq Ct score > 28, low percentage of total RNA reads compared to the percentage of total DNA reads, < 20.000 RNA unique fragments and < 30% RNA unique fragments, and/or Fusion QC score < 10 (Suppl. Table S4). A total of nine samples (#7, #8, #10, #11, #12, #14, #15, #16 and #31) were found ALK-positive by IHC and/or FISH. One sample (#13) showed discordant IHC and FISH results (ALK 2+ by IHC while found negative by FISH). One sample (#9) had a doubtful IHC results while found borderline by FISH (13% of cells with split signals or single fluorescent red signals). These analyses were not repeated due to lack of material available. One sample (#8) had a positive IHC results while found inconclusive by FISH. Three samples (#6, #17 and #18) were identified as ROS1-positive by IHC and FISH, and 2 samples (#22 and #30) had inconclusive results.

**Table 5:** Molecular characteristics of the 35 FFPE tumour samples analysed by the standard procedures (RNASeq, *ALK* and *ROS1* IHC, and *ALK* and *ROS1* FISH).

ID sample	Tumour content (%)	Specimen type	RNASeq (genes implied, allelic frequency and gene fusion details)	ALK IHC	ALK FISH	ROS1 IHC	ROS1 FISH
1	25	SR	No fusion	0	–	0	–
2	15	SR	No fusion	0	–	1+	Negative
3	70	SR	No fusion	0	–	0	–
4	75	SR	No fusion	0	–	0	–
5	30	SR	No fusion	0	–	0	–
6	60	SR	No fusion	0	–	1+	Positive
7	70	Biopsy	EML4 (exon 2) :: ALK(exon 20) 96% (chr2:42472827; chr2:29446394)	2+	Positive	0	–
8	25	SR	EML4 (exon 2) :: ALK (exon 20) 96% (chr2:42472827; chr2:29446394)	3+	Inconclusive	2+	Negative
9	40	SR	EML4 (exon7):: ALK (exon20) 68% (chr2:42492091; chr2:29446394)	2+	Negative (13%)	0	–
10	60	Biopsy	EML4 (exon6) :: ALK (exon 20) 74% (chr2:42491871; chr2:29446394)	2+	Positive	0	–
11	30	Biopsy	EML4 (exon 7) :: ALK (exon 20) 75% (chr2:42492091; chr2:29446394)	1+	Positive	0	–
12	60	Biopsy	EML4 (exon18) :: ALK (exon 20) 98% (chr2:42543190; chr2:29446394)	3+	Positive	2+	Negative
13	40	SR	EML4 (exon 13) :: ALK (exon 20) 96% (chr2:42522656; chr2:29446394)	3+	Negative	3+	Negative
14	10	Cytology sample	EML4 (exon 13) :: ALK (exon 20) 92% (chr2:42522656; chr2:29446394)	1+	Positive	1+	Negative
15	70	Biopsy	EML4 (exon 13) :: ALK (exon 20) 98% (chr2:42522656; chr2:29446394)	1+	Positive	0	–
16	70	Biopsy	EML4 (exon 20) :: ALK (exon 20) 99% (chr2:42552694; chr2:29446394)	3+	–	0	–
17	65	Biopsy	CD74 (exon 6) :: ROS1 (exon 34) 90% (chr5:149784243; chr6:117645578)	0	–	3 +	positive
18	85	Biopsy	SDC4 (exon 2) :: ROS1 (exon 32) 83% (chr20:43964422; chr6:117650609)	0	–	3 +	positive
19	30	Biopsy	KIF5B (exon 15) :: RET (exon 12) 91% (chr10:32317356; chr10:43612032)	0	–	0	–
20	25	SR	MET (exon 13) :: MET (exon 15) 74% (chr7:116411708; chr7:116414935)	0	–	0	–
21	30	SR	MET (exon 13) :: MET (exon 15) 92% (chr7:116411708; chr7:116414935)	0	–	0	–
22	15	Biopsy	MET (exon 13) :: MET (exon 15) 79% (chr7:116411708; chr7:116414935)	0	–	1+	Inconclusive
23	15	SR	MET (exon 13) :: MET (exon 15) 58% (chr7:116411708; chr7:116414935)	0	–	0	–
24	50	SR	Low sequencing quality but reasonable suspicion of *MET* exon 14 skipping MET (exon 13) :: MET (exon 15) 50% (chr7:116411708; chr7:116414935)	0	–	0	–
25	60	SR	Inconclusive	0	–	0	–
26	30	SR	Inconclusive	0	–	0	–
27	15	SR	Inconclusive	0	–	0	–
28	80	SR	Inconclusive	0	–	0	–
29	50	SR	Inconclusive	0	–	0	–
30	60	SR	Inconclusive	0	–	1+	Inconclusive
31	45	SR	Inconclusive	3+	Positive	2+	Negative
32	70	SR	Inconclusive	0	–	1+	Negative
33	30	Biopsy	Inconclusive	0	–	1+	Negative
34	25	Biopsy	Inconclusive	0	–	2+	Negative
35	50	cytology sample	Inconclusive	0	–	2+	Negative

– : analysis not performed, *FISH* fluorescent in-situ hybridization, *IHC* immunohistochemistry, *RNASeq* RNA-based sequencing, *SR* surgical resection.

### Data analysis

The performance of the Idylla GeneFusion assay was evaluated based on the determination of *ALK, ROS1, RET, NTRK* and *MET* status in 35 FFPE tumour specimens, using IHC/FISH or RNASeq as the reference. Overall agreement is calculated as the number of samples with concordant status between the tested assay and the reference method out of the overall number of samples analysed with valid results. Sensitivity is defined as the proportion of rearranged or MET-altered samples obtained by the tested assay among the rearranged / MET-altered samples according to the reference method. Specificity represents the proportion of non-rearranged/non MET-altered samples obtained by the tested assay among the non-rearranged/non-MET altered samples according to the reference method. Positive predicted value (PPV) is calculated as the proportion of true positive results among all positive results obtained by the tested assay. Negative predicted value (NPV) is calculated as the proportion of true negative results among all negative results obtained by the tested assay. A 95% confidence interval (95% CI) was calculated for sensitivity and specificity using Wilson's method^42, 43^.

### Limits of detection (LOD) of the Idylla GeneFusion assay

Two different commercial fusion RNA standards were used to determine the limit of detection of the Idylla GeneFusion assay: the Seraseq^®^ FFPE Tumor Fusion RNA v4 Reference Material (LGC Seracare) and the Horizon ALK-RET-ROS1 Fusion FFPE RNA Reference Standard (reference HD784, Horizon Discovery Ltd, Waterbeach, UK). The specific gene fusions covered by the two standards are detailed in Suppl. Tables S3 and S5. Both consist in one 10 µm-thick FFPE section prepared from cell lines with characterized gene fusions and exon skipping events. For each standards, RNA extraction was performed using the RNeasy FFPE kit (Qiagen). RNA concentrations were determined using the Qubit 3 fluorometer and the Qubit RNA HS Assay kit (Thermo Fisher Scientific). Twenty microliter solutions with different RNA inputs were loaded into the lysis pad of the cartridges prior the launch of the analyses. The LOD was determined for each of the alterations as the lowest input yielding a positive result by Idylla.

### Supplementary Information

Supplementary Tables.

## Data Availability

The data generated during the current study are available from the corresponding author on reasonable request.
