# Supplementary material for: Validation of the Idylla GeneFusion assay to detect fusions and MET exon-skipping in non-small cell lung cancers
Source: Sci Rep. 2023 Aug 9;13:12909. doi: 10.1038/s41598-023-39749-4 (PMC10412571; doi:10.1038/s41598-023-39749-4)
Supplement: Supplementary file 1 — Supplementary Tables. [file 41598_2023_39749_MOESM1_ESM.docx]

**Supplementary Tables**

**Supplementary Table S1.** Specific oncogenic alterations detected by the Idylla GeneFusion assay

| Gene | Number of alterations detected | Type of alterations detected | Fusion partners | Details on the oncogenic alterations detected |
| --- | --- | --- | --- | --- |
| ALK | 16 | Gene fusions | EML4 | EML4 (exon 2) :: ALK (exon 20) |
|  |  |  |  | EML4 (exon 6) :: ALK (exon 20) |
|  |  |  |  | EML4 (exon 13) :: ALK (exon 20) |
|  |  |  |  | EML4 (exon 15) :: ALK (exon 20) |
|  |  |  |  | EML4 (exon 17) :: ALK (exon 20) |
|  |  |  |  | EML4 (exon 18) :: ALK (exon 20) |
|  |  |  |  | EML4 (exon 20) :: ALK (exon 20) |
|  |  |  | KIF5B | KIF5B (exon 15) :: ALK (exon 20) |
|  |  |  |  | KIF5B (exon 17) :: ALK (exon 20) |
|  |  |  |  | KIF5B (exon 24) :: ALK (exon 20) |
|  |  |  | HIP1 | HIP1 (exon 28) :: ALK (exon 20) |
|  |  |  |  | HIP1 (exon 30) :: ALK (exon 20) |
|  |  |  | KLC1 | KLC1 (exon 9) :: ALK (exon 20) |
|  |  |  | TPR | TPR (exon 15) :: ALK (exon 20) |
|  |  |  | TFG | TFG (exon 4) :: ALK (exon 20) |
|  |  |  |  | TFG (exon 6) :: ALK (exon 20) |
| ROS1 | 13 |  | CD74 | CD74 (exon 6) :: ROS1 (exon 32) |
|  |  |  |  | CD74 (exon 6) :: ROS1 (exon 34) |
|  |  |  | SDC4 | SDC4 (exon 2) :: ROS1 (exon 32) |
|  |  |  |  | SDC4 (exon 4) :: ROS1 (exon 32) |
|  |  |  |  | SDC4 (exon 4) :: ROS1 (exon 34) |
|  |  |  | SLC34A2 | SLC34A2 (exon 4) :: ROS1 (exon 32) |
|  |  |  |  | SLC34A2 (exon 4) :: ROS1 (exon 34) |
|  |  |  |  | SLC34A2 (exon 13) :: ROS1 (exon 32) |
|  |  |  | EZR | EZR (exon 10) :: ROS1 (exon 34) |
|  |  |  | TPM3 | TPM3 (exon 8) :: ROS1 (exon 35) |
|  |  |  | GOPC | GOPC (exon 4) :: ROS1 (exon 36) |
|  |  |  |  | GOPC (exon 8) :: ROS1 (exon 35) |
|  |  |  | LRIG3 | LRIG3 (exon 16) :: ROS1 (exon 35) |
| RET | 7 |  | KIF5B | KIF5B (exon 15) :: RET (exon 11) |
|  |  |  |  | KIF5B (exon 15) :: RET (exon 12) |
|  |  |  |  | KIF5B (exon 16) :: RET (exon 12) |
|  |  |  |  | KIF5B (exon 22) :: RET (exon 12) |
|  |  |  |  | KIF5B (exon 23) :: RET (exon 12) |
|  |  |  |  | KIF5B (exon 24) :: RET (exon 11) |
|  |  |  | CCDC6 | CCDC6 (exon 1) :: RET (exon 12) |
| MET | / | Exon skipping | / | MET (exon 13) :: MET (exon 15) |

**Supplementary Table S2.** Regions of interest in genes analysed by targeted RNASeq approach for the detection of gene fusions and exon skipping alterations.

| Genes | Reference sequences | Regions of interest |
| --- | --- | --- |
| ALK | NM_004304.5 | exons 2, 4, 6, 10, 16, 17, 18, 19, 20, 21, 22, 23 and 26 (5’) |
| BRAF | NM_004333.6 | exons 2, 7, 8, 9, 10, 11, 12, 15 and 16 (5’)  exons 1, 3, 7, 8, 10 and 13 (3’) |
| EGFR | NM_005228.5 | exons 7, 8, 9, 16, 19 and 20 (5’)  exons 1, 24 and 25 (3’) |
| FGFR1 | NM_015850.4 | exons 2, 3, 4, 5, 6, 7, 8, 9, 10, 11 and 17 (5’)  exons 12 and 17 (3’) |
| FGFR2 | NM_000141.5 | exons 2, 5, 7, 8, 9 and 10 (5’) exons 16 and 17 (3’) |
| FGFR3 | NM_000142.5 | exons 3, 5, 8, 9 and 10 (5’) exons 16, 17 and 18 (3’) |
| MET | NM_000245.4 | exons 2, 4, 5, 6, 13, 14, 15, 16, 17 and 21 (5’)  exons 2 and 13 (3’) |
| NRG1 | NM_013957.5  NM_004495.4  NM_013962.3 | exons 1 and 8 (5’)  exons 1, 2, 3, 4 and 6 (5’)  exon 1 (3’) |
| NTRK1 | NM_002529.4 | exons 2, 4, 6, 8, 10, 11, 12 and 13 (5’) |
| NTRK2 | NM_006180.6 | exons 5, 7, 9, 11, 12, 13, 14, 15, 16 and 17 (5’) |
| NTRK3 | NM_002530.4  NM_001007156.3 | exons 4, 7, 10, 12, 13, 14, 15 and 16 (5’)  exons 13, 14 and 15 (3’)  exon 15 (5’) |
| RET | NM_020630.6  NM_020975.6 | exons 2, 4 and 6 (5’)  exons 8, 9, 10, 11, 12, 13 and 14 (5’) |
| ROS1 | NM_002944.3 | exons 2, 4, 7, 31, 32, 33, 34, 35, 36 and 37 (5’) |

**Supplementary Table S3.** Details on the specific gene fusions covered by the commercial Seraseq FFPE Tumor Fusion RNA v4 Reference Material (LGC Seracare).

RNA concentrations: 47.2 ng/µL

| Gene fusions | Expected allelic frequence (% reads) |
| --- | --- |
| CCDC6 (exon 1) :: RET (exon 12) | **37** |
| CD74 (exon 6) :: ROS1 (exon 34) | **72** |
| EGFR (exon 1) :: EGFR (exon 8) | 97 |
| EGFR (exon 24) :: SEPT14 (exon 10) | 98 |
| EML4 (exon 13) :: ALK (exon 20) | **97** |
| ETV6 (exon 5) :: NTRK3 (exon 15) | **96** |
| FGFR3 (exon 17) :: BAIAP2L1 (exon 2) | 36 |
| FGFR3 (exon 17) :: TACC3 (exon 11) | 65 |
| KIF5B (exon 24) :: RET (exon 11) | **99** |
| LMNA (exon 2) :: NTRK1 (exon 10) | **33** |
| MET (exon 13) :: MET (exon 15) | **96** |
| NCOA4 (exon 8) :: RET (exon 12) | 38 |
| PAX8 (exon 9) :: PPARG1 (exon 3) | ND |
| SLC34A2 (exon 4) :: ROS1 (exon 34) | **28** |
| SLC45A3 (exon 1) :: BRAF (exon 8) | 19 |
| TFG (exon 5) :: NTRK1 (exon 9) | **47** |
| TMPRSS2 (exon 1) :: ERG (exon 2) | ND |
| TPM3 (exon 7) :: NTRK1 (exon 9 ) | **55** |

Lines in bold type indicate alterations that should be covered by the idylla GeneFusion assay.

**Supplementary Table S4.** Details on sequencing quality for the 11 clinical samples (#25 to #35) with inconclusive results due to insufficient RNA quality.

| ID sample | Pre-Seq Ct score | Percentage of RNA reads compared to the percentage of DNA reads | Percentage of RNA reads | Number of RNA unique fragments | Percentage of RNA unique fragments | Fusion QC score (unique RNA start sites per GSP2 control) |
| --- | --- | --- | --- | --- | --- | --- |
| 25 | 28 | < DNA reads | 3.3 | 2817 | 3% | 9.38 |
| 26 | 30 | < DNA reads | 3.7 | 1687 | 3.6% | 7 |
| 27 | 29 | < DNA reads | 20.4 | 1780 | 17.9% | 7 |
| 28 | 30 | < DNA reads | 2.3 | 3340 | 2.4% | 2.25 |
| 29 | 30 | < DNA reads | 5.1 | 532 | 5% | 1.62 |
| 30 | 30 | < DNA reads | 34.6 | 2831 | 31.5% | 4.12 |
| 31 | 30 | < DNA reads | 6.1 | 906 | 5.6% | 1.75 |
| 32 | 30 | > DNA reads | 41.2 | 857 | 34.7% | 4.75 |
| 33 | 30 | < DNA reads | 5.3 | 52 | 5.9% | 0.62 |
| 34 | 30 | < DNA reads | 21.5 | 2379 | 14.5% | 9 |
| 35 | 30 | < DNA reads | 9.8 | 370 | 7.2% | 1.75 |

Sequencing quality was assessed by the following metrics: a minimum of 500,000 total reads per sample*, an on-target > 85%*, a percentage of total RNA reads over the percentage of total DNA reads, a minimum of 20.000 reads and 30% reads for RNA unique fragments, an average unique RNA start sites per GSP2 control (Fusion QC score) > 10, a RNA median fragment length > 100 base pairs*.

*The total number of reads per sample, the percentage of on-target and the RNA median fragment length were over the cut-off values for all the 12 clinical samples.

**Supplementary Table S5.** Details on the specific gene fusions covered by the commercial Horizon ALK-RET-ROS1 Fusion FFPE RNA Reference Standard (Horizon Discovery Ltd).

RNA concentrations: 64.5 ng/µL

| Gene fusions | Expected allelic frequency  (% reads) |
| --- | --- |
| **EML4 (exon 13) :: ALK (exon 20)** | **95** |
| **CCDC6 (exon 1) :: RET (exon 12)** | **72** |
| **SLC34A2 (exon 4) :: ROS1 (exon 32)** | **95** |

Lines in bold type indicate alterations that should be covered by the idylla GeneFusion assay.
